# Sensor-Based and Patient-Based Assessment of Daily-Living Physical Activity in People with Parkinson’s Disease: Do Motor Subtypes Play a Role?

**DOI:** 10.3390/s20247015

**Published:** 2020-12-08

**Authors:** Irina Galperin, Talia Herman, Mira Assad, Natalie Ganz, Anat Mirelman, Nir Giladi, Jeffrey M. Hausdorff

**Affiliations:** 1 Center for the Study of Movement, Cognition and Mobility, Neurological Institute, Tel Aviv Sourasky Medical Center, Tel Aviv 6492415, Israel; irag@tlvmc.gov.il (I.G.); talih@tlvmc.gov.il (T.H.); miraassad@mail.tau.ac.il (M.A.); natalieza@tlvmc.gov.il (N.G.); anatmi@tlvmc.gov.il (A.M.); nirg@tlvmc.gov.il (N.G.); 2 Department of Physiology and Pharmacology, Sackler Faculty of Medicine, Tel Aviv University, Tel-Aviv 69978, Israel; 3 Sagol School of Neuroscience, Tel Aviv University, Tel-Aviv 69978, Israel; 4 Department of Neurology and Neurosurgery, Sackler School of Medicine, Tel Aviv University, Tel-Aviv 69978, Israel; 5 Department of Physical Therapy, Sackler Faculty of Medicine, Tel-Aviv 69978, Israel; 6 Rush Alzheimer’s Disease Center and Department of Orthopedic Surgery, Rush University Medical Center, Chicago 60612, IL, USA

**Keywords:** wearables, accelerometer, physical activity, Parkinson’s disease, motor subtypes

## Abstract

The benefits of daily-living physical activity are clear. Nonetheless, the relationship between physical activity levels and motor subtypes of Parkinson’s disease (PD), i.e., tremor dominant (TD) and postural instability gait difficulty (PIGD), have not been well-studied. It is also unclear if patient perspectives and motor symptom severity are related to objective, sensor-based assessment of daily-living activity in those subtypes. To address these questions, total daily-living physical activity was quantified in 73 patients with PD and 29 healthy controls using a 3D-accelerometer worn on the lower back for at least three days. We found that individuals with the PIGD subtype were significantly less active than healthy older adults (*p* = 0.007), unlike individuals with the TD subtype. Among the PIGD subtype, higher daily physical activity was negatively associated with more severe ON bradykinesia (r_S_ = -0.499, *p* = 0.002), motor symptoms (higher ON MDS-UPDRS (Unified Parkinson’s Disease Rating Scale motor examination)-III scores), gait difficulties (r_S_ = -0.502, *p* = 0.002), motor complications (r_S_ = 0.466, *p* = 0.004), and balance (r_S_ = 0.519, *p* = 0.001). In contrast, among the TD subtype, disease-related characteristics were not related to daily-living physical activity. Intriguingly, physical activity was not related to self-report of ADL difficulties (scores of the MDS-UPDRS Parts I or II) in both motor subtypes. These findings highlight the importance of objective daily-living physical activity monitoring and suggest that self-report does not necessarily reflect objective physical activity levels. Furthermore, the results point to important differences in factors related to physical activity in PD motor subtypes, setting the stage for personalized treatment programs.

## 1. Introduction

The positive benefits of a daily-living physical activity on health in general, and motor function, in particular, and the negative consequences of reduced physical activity have been well-described [1,2]. Emerging evidence highlights the importance of capturing daily-living physical activity metrics with activity monitoring via body-worn sensors. Signals from an activity monitor, typically placed on the lower-back or wrist, can be converted into metrics that reflect the intensity and total daily-living activity. This approach mitigates problems of self-report, like recall bias and other sources of subjectivity [3,4,5,6].

Previous studies among patients with Parkinson’s disease (PD) have shown, not surprisingly, that objectively measured, sensor-based estimates of daily-living physical activity are reduced, compared to that seen in age-matched controls [3]. Moreover, a prolonged reduction in physical activity is related to increased severity of motor symptoms [4]. In addition, differences between the two common motor subtypes, i.e., postural instability and gait difficulty (PIGD) and the tremor dominant (TD) subtypes, have been well studied. However, previous work has examined this issue either via self-report or semi-objective tests inside the laboratory settings [7,8]. Daily-lining physical activity based on objective sensor-based assessments in the two sub-types is not yet clear. One could speculate that physical activity would be lower among PD patients with the PIGD motor subtype, compared to those with the TD subtype. Indeed, balance and objective measures of gait extracted from accelerometer-based wearable sensors (McRoberts, DynaPort Hybrid system, The Netherlands) in laboratory settings were worse in the PIGD patients, compared to TD patients [6]. Nonetheless, contrary to expectations, no difference was found between PIGD and TD subtypes in daily-living step counts—an important component of physical activity, extracted from activity monitoring continuously over the course of several days [4]. However, total daily-living physical activity was not examined in that study. Furthermore, the total daily-living physical activity was not compared to a healthy adult control group, limiting the ability to interpret these findings. Comparing daily-living activity of healthy adults to those measured in individuals of the PIGD and the TD subtype might provide a more accurate reflection of the impact of PD on the everyday life of these two motor subtypes. This may, perhaps, provide insight into the personalization of treatment.

In PD, especially in the PIGD subtype, walking is affected by the burden of motor symptoms, yet partially improved by levodopa medication intake. Therefore, one could speculate that motor severity during the ON medication state should be more closely associated with daily-living physical activity than motor severity measured during the OFF medication state. Nonetheless, one study reported no differences in the amount of walking captured by a smartwatch accelerometer during self-reported ON medication state and during wearing off in mild to moderately affected people with PD [9,10]. In contrast, another study reported moderate correlations between physical activity and motor symptoms observed both in the ON and the OFF state [10]. However, the relationship between physical activity and motor disease severity during the ON and OFF medication cycles was not examined separately in individuals from the PIGD and the TD subtypes.

Previous studies suggest that there is a mild-to-moderate association between motor symptom severity (e.g., Unified Parkinson’s Disease Rating Scale motor examination score(MDS-UPDRS)-III) in the ON state and daily-living physical activity [10,11,12]. At the same time, the understanding of how other different features of the MDS-UPDRS are associated with daily-living physical activity in PD is still lacking. For instance, patient perspectives (self-report) of the impact of non-motor and motor symptoms on daily living experiences could be expected to be associated with objectively measured, sensor-based assessment of daily-living physical activity. Indeed, the MDS-UPDRS Part II has been suggested to be an indicator of disability [13], and increasingly, regulatory agencies are underscoring the importance of the perspective of the patient. However, it is not yet clear if and how self-report estimates of physical activity reflect objective, sensor-based assessment. We aimed to shed light on this question by comparing self-report to objectively measured physical activity among individuals with PD. Further, one could suggest that patients who spend much of their time in the off-medication state (more severe motor complications, as reflected in the MDS-UPDRS Part IV) would have lower daily-living physical activity levels. Scores on the MDS-UPDRS Part III and Part IV have been correlated with acceleration measured under free-living conditions [11,12]. In addition, an association between motor fluctuations (self-reported) and time spent walking during the day was established; however, the severity of fluctuations was not associated with the amount of change in time spent walking in relation to levodopa intake in any part of the day [9,11,12]. We conjectured that motor fluctuation severity (MDS-UPDRS Part IV) would be, in a way, related to an objective measure of total daily-living physical activity during the day in individuals of both motor subtypes. Moreover, we speculated that in the PIGD subtype who have more severe changes in mobility, this association would be stronger.

To gain additional insight into daily-living physical activity among patients with the common motor subtypes of PD, we tested the following hypotheses: (1) Sensor-based assessment of total daily-living physical activity is lower in subjects with the PIGD motor subtype, compared to those with the TD subtype and compared to healthy controls (HC); (2) disease severity in the ON-medication state is associated with daily-living physical activity, and more than the association in the OFF-medication state, and (3) higher self-reported difficulty of non-motor and motor experiences of daily living (i.e., MDS-UPDRS Parts I, II and IV) are related to lower levels of daily physical activity. To our surprise, the results below are only consistent with a subset of these hypotheses.

## 2. Materials and Methods

### 2.1. Study Design and Setting

This cross-sectional study was undertaken in the Center for the study of Movement, Cognition, and Mobility (CMCM) at the Tel Aviv Sourasky Medical Center, Tel Aviv, Israel.

### 2.2. Participants

The study was approved by the Tel Aviv Medical Center Ethics Committee (0595-09-TLV; TLV-0674-15, NIH: NCT01039831). All subjects provided informed written consent for the study according to the Declaration of Helsinki.

This work is based on the secondary analysis of data that was originally collected to examine the association between white matter hyperintensities and PD motor subtypes [14]. A full description of participant recruitment and demographics was reported elsewhere [6]. Briefly, patients with PD between the age of 40 and 80 years and with disease severity of stage I–IV on the Hoehn and Yahr scale were included. Patients were excluded if they were demented, based on the DSM-IV criteria and Mini Mental State Exam (MMSE) scores, or if they underwent brain surgery. Subjects were also excluded if they had significant co-morbidities likely to affect gait, e.g., acute illness, orthopedic disease, history of stroke, or if they could not walk independently in the off medication cycle. For the comparison of the daily living physical activity among PD subtypes and healthy adults, we included data from a healthy control (HC) group. The HC underwent gait-lab and daily-living evaluation between September 2017 and December 2019 as part of a study designed to examine the association of motor, behavioral, psycho-cognitive, and other factors with total daily-living physical activity in HC and PD patients. Briefly, HC participants were between the ages of 60 and 85 years without any neurological, neuropsychiatric, orthopedic diagnosis, dementia, or major depression.

### 2.3. Demographic and Clinical Measures

Demographics and subject characteristics were collected from the entire cohort. Parkinsonian symptoms and disease severity were evaluated using the Unified Parkinson’s Disease Rating Scale-new version, the MDS-UPDRS [15]. The MDS-UPDRS was first assessed in the OFF medication state (12 h after taking anti-parkinsonian medications). Subsequently, patients took their morning medications, and the evaluation continued in the ON state, reflecting their performance while on dopaminergic therapy. Patients were classified into PIGD or TD subtypes (in OFF medication state) [6,14]. For classification into PD motor subtypes in the off state, items from parts 2 and 3 of the original UPDRS were used, as suggested by Jankovic et al. [16]. Bradykinesia of the lower extremities was calculated from items 3.7 and 3.8 of the motor MDS-UPDRS part. A gait index score was calculated as the sum of 4 items related to gait (3.9-12).

The levodopa equivalent daily dose (LEDD) scores were calculated [17]. The clinical examination included cognitive, gait, balance, and function tests [6,14]. Cognition was evaluated using the Mini Mental State Exam (MMSE) and the Montreal Cognitive Assessment (MoCA) tests. Balance was measured using the Berg balance scale test and mental well-being was assessed via the Geriatric Depression Scale (GDS-15). The severity of freezing of gait was assessed by self-report using the NFOG-Q [18]. The Activities-specific Balance Confidence scale (ABC) evaluated the level of fear of falling while walking.

### 2.4. Test Protocol

After testing in the lab, participants were asked to wear a small, lightweight body-fixed sensor (DynaPort Hybrid system, The Netherlands) attached with a belt to their lower back for three consecutive days (except during activities like showering). The hybrid contains a triaxial accelerometer and a tri-axial gyroscope (data not analyzed in the present study). The three acceleration axes were: Vertical (V), medio-lateral (ML), and anterior posterior (AP). To measure daily-living physical activity among the healthy controls, participants wore a similar tri-axial accelerometer (Axivity AX3, York, UK) for one week. The device was held in place with skin tape [19]. In order to evaluate “whole body” movement, both devices were placed close to the center of mass, as previously described by Mathie et al. [20] and elsewhere [21], and used in other studies [22,23,24] (among many others). All subjects were instructed to continue their daily routine. Upon completion of the recording, the devices were returned to the laboratory for data processing. Subjects who had less than three days of recording and less than 60 percent wear-time were excluded.

### 2.5. The Total Daily-Living Physical Activity Counts

The raw acceleration signals were sampled at 100 Hz, saved locally on the sensors, and later transferred to a personal computer for further analysis using MATLAB (MathWorks, Natick, MA, USA) software. The data was extracted from 100 Hz raw tri-axial acceleration data after calibration, removal of gravity and sensor noise, and identification of wear/non-wear episodes. Then, the signal vector magnitude (SVM) of the measured acceleration signal from three axes, calculated for each 15 s window during the daytime (between 06:00 AM and 22:00 PM each day):

SVM = sqrt (x^2^ + y^2^ + z^2^) (1)

Per each day, the sum of all windows was calculated and normalized by the wear time in hours. Finally, the median value of all days was extracted for the sum values for each subject. This parameter represents total daily-living physical activity (physical activity), as previously described [20] and has been widely used in studies of healthy adults and individuals with PD [1,12,25,26] We also divided the SVM counts per 100 to simplify the counts (mg).

### 2.6. Statistical Analyses

Statistical analyses were carried out using SPSS v25 (SPSS Inc, Chicago, IL, USA). Descriptive statistics (median/means and range/SD) were calculated for subject characteristics. To compare the PIGD and the TD subtypes, Mann–Whitney U and Student t-tests were used. To compare subject characteristics, Kruskal–Wallis tests were used. Univariate General Linear Model (GLM), controlling for age, sex, and body-mass index (BMI) with 95% confidence intervals examined the relationship between the three groups in total daily-living activity counts. Since only one subject from the PIGD subgroup remained in stage 4 (ON medication) in an exploratory analysis, we removed this subject from the PIGD subgroup. The decision to control for age, sex, and BMI was due to the previously reported association between these subject characteristics and total daily physical activity [25]. Since the subjects from the control group were older than the PD subjects, all statistical analyses were controlled for age. Moreover, as age could be a confounding variable, we also examined potential interaction effects between age and total daily physical activity. Post Hoc Multiple Comparisons analysis was carried out using Fisher’s least significant differences (LSD). To avoid false-positive findings due to multiple comparisons, we used Bonferroni corrections. According to the Bonferroni corrected LSD, comparisons are significant if the respective *p*-values are below 0.017 (0.05/3 groups). Finally, we used Spearman’s partial correlations to examine the relationship between disease-related characteristics and daily-living physical activity in the PD subtypes. Due to the association between age and BMI with physical activity, and the association of sex with disease severity, Spearman’s correlation analyses adjusted to these factors. According to the Bonferroni correction, significance levels were set at *p* = 0.0033 (0.05/15 outcomes).

## 3. Results

Participants from the PIGD and TD subgroups had similar demographic characteristics. Of note, individuals in the control group (HC) were significantly older (about 5 years) than the subjects on the PD subtypes (see Table 1). Most of the participants lived in urban areas (88–91%). Of all participants, 72.0% up to 80.4% were married. Among the HC as well as the TD, 31.0% and 34.0% respectively were working (or volunteering). Interestingly, a significantly higher employment rate was observed in the PIGD subgroup (*p* = 0.010, 65.0% reported working or volunteering). The PIGD and TD subgroups were generally similar with respect to disease-related characteristics (e.g., disease duration, UPDRS motor score, levodopa equivalent daily dose), except for higher scores in the MDS-UPDRS Part I and in the PIGD as compared to the TD subgroup. Geriatric Depression Scale (GDS-15) scores were similar in both PD subtypes and suggested the absence of major depression.

**Figure 1:**
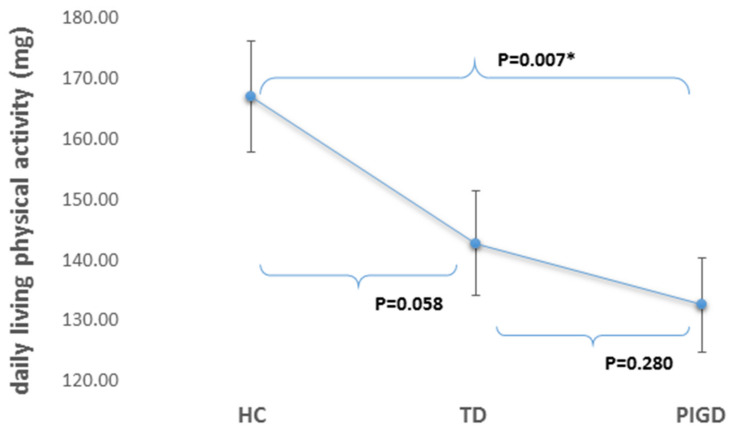
Daily-living physical activity counts (*Y*-axis) in PIGD, TD, and healthy control (HC) (*X*-axis), controlling for age, sex, and BMI. * Difference statistically significant (*p* < 0.017) even after adjusting for multiple comparisons using Bonferroni corrections. The interaction age *group* body-mass index (BMI) was significant (*p* < 0.001) and the associations with group persisted (*p* = 0.007) also after removing the only subject with H&Y stage 4 in ON from the PIGD subgroup (*p* = 0.011).

Daily-living physical activity was significantly lower (*p* = 0.007) in the PIGD subgroup 166.97 ± 9.19 (mg) as compared to the HCs 132.57 ± 7.87 (mg) 95% CI [−61.570, −8.861] (see Figure 1). Physical activity tended to be different in the TD subgroup (142.75 ± 8.65 mg), compared to PIGD 95% CI [−16.210, 32.68] and the HC 95% CI [−53.517, −0.447], although this was not statistically significant after controlling for multiple comparisons. After removing one subject (H&Y stage 4 in ON) from the PIGD subgroup, the differences between the HC and PIGD remained significant (*p* = 0.011), 95% CI [−61.775,−8.252], and non-significant for HC and TD (*p* = 0.064) after adjusting to multiple comparison, 95% CI [−53.73,−0.148]. In addition, an exploratory analysis of interactions between age and other variables (e.g., physical activity, group, gender) only slightly changed the *p*-value (however, findings remained significant).

Among the entire cohort (PD and HC), higher age (r_S_ = −0.290, *p* = 0.003), and higher BMI (r_S_ = −0.269, *p* = 0.007) were mildly correlated with lower levels of daily-living physical activity. Gender was not associated with daily-living physical activity (*p* > 0.05). Cognitive function, as represented by the MoCA scores, was related to total daily physical activity in the HC group (r_S_ = 0.413, *p* = 0.026) but not among all the participants with PD (*p* > 0.05) nor in each subtype separately (*p* > 0.05). Also, mood disorders (GDS-15 scores) were not associated with the physical activity levels in PD subjects (r_S_ = −0.055, *p* = 0.641).

**Figure 2:**
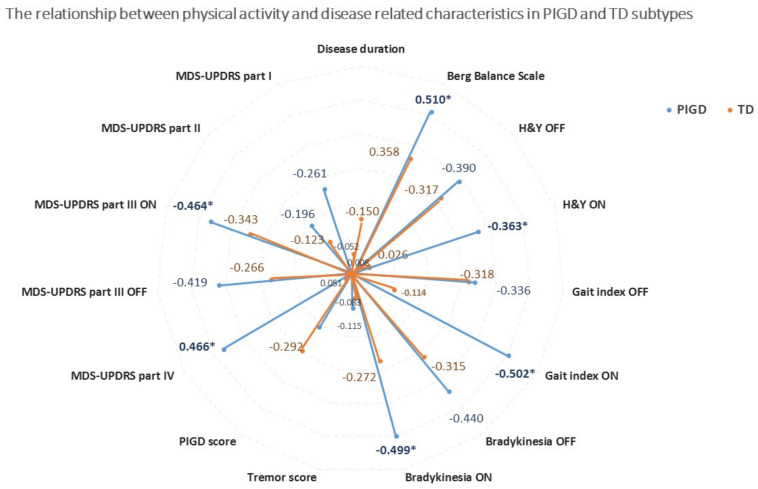
The relationship between total daily-living physical activity and disease-related measures in PIGD and TD subtypes. Blue indicates PIGD and orange indicates TD. The values represent the Spearman partial correlation coefficient (r_S_) controlling for age, sex, and BMI. The correlation coefficients are presented as absolute values. * *p*-value statistically significant (*p* < 0.004), even after adjusting for multiple comparisons using Bonferroni corrections.

Figure 2 summarizes the association between total daily-living physical activity and disease-related factors in the TD and the PIGD subtypes. Among the PIGD subjects, higher physical activity was negatively associated with more severe motor symptoms (higher scores UPDRS Part III) in ON (r_S_ = −0.464, p = 0.004) and in OFF (r_S_ = −0.419, p = 0.011) medication states, and with motor complications (UPDRS Part IV score) (r_S_ = 0.466, p = 0.004). Conversely, these associations were not observed in the TD subtype.

**Figure 3:**
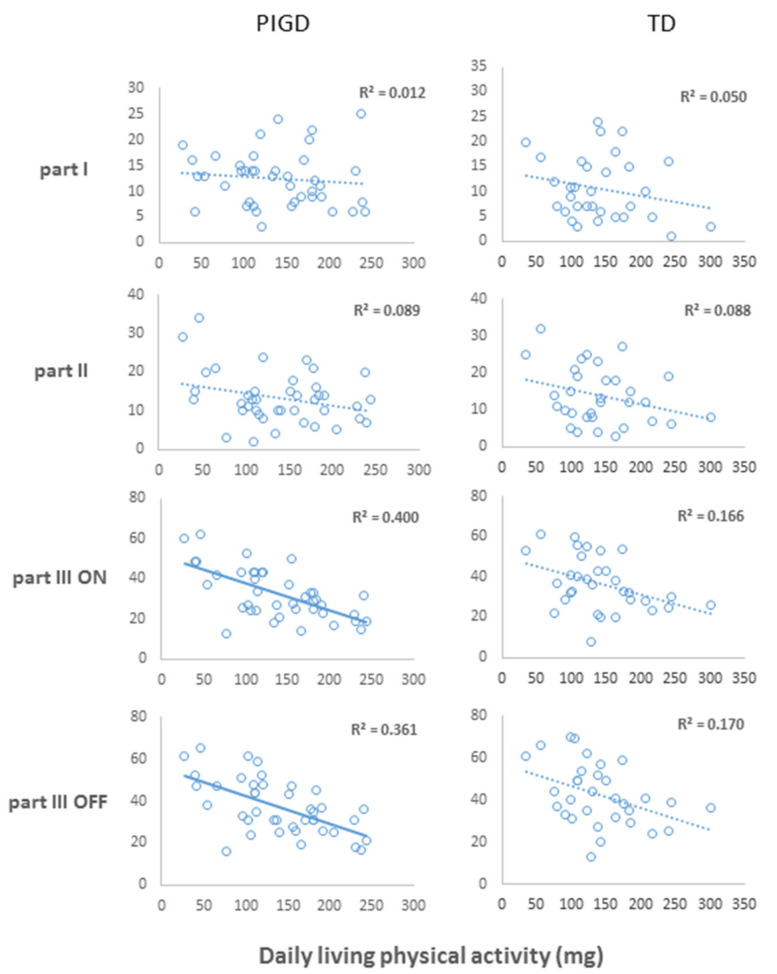
The relationship between total daily-living physical activity and MDS-UPDRS Parts in PIGD and TD. Scatter-plots illustrating the relationship of daily-living physical activity extracted from acceleration signals (*x*-axis) against the MDS-UPDRS Parts scores (*y*-axis) in PIGD and TD subtypes. Regression lines are included with solid lines showing *p*-value < 0.005 (0.05/10 outcomes), dashed lines *p*-value < 0.005. The *p*-values represent the variable’s eta significance in linear regressions controlled by age, sex and BMI. MDS-UPDRS—Unified Parkinson’s Disease Rating Scale motor examination.

Daily-living physical activity was related to the lower extremity bradykinesia scores, measured in ON medication state in the PIGD group (r_S_ = −0.499, *p* = 0.002). However, the lower extremity bradykinesia score in the OFF medication state had only a slightly weaker association (r_S_ = −0.440, *p* = 0.007) with physical activity (see Figure 2). The gait index in ON was significantly (r_S_ = −0.502, *p* = 0.002) associated with physical activity only in the PIGD group. Conversely, in the TD subtype, no significant association was found between disease-related characteristics and daily physical activity. MDS-UPDRS total score in ON was moderately and significantly correlated with physical activity as well (r_S =_ −0.455, *p* = 0.005) in the PIGD group, but not in the TD group (r_S =_ −0.175, *p* = 0.374). No associations were found between daily physical activity and the MDS-UPDRS scores of Parts I (r_S_ = −0.261, *p* = 0.125; r_S_ = −0.052, *p* = 0.793) or II (r_S_ = −196, *p* = 0.251; r_S_ = −0.125, *p* = 0.532) among the PIGD and the TD subtypes, respectively (see Figure 3).

Daily-living physical activity was associated with better balance as represented by higher Berg Balance Test scores in the PIGD subtype (r_S_ = 0.519, *p* = 0.001) and in the TD subtype (r_S_ = 0.358, *p* = 0.044). Moreover, daily-living physical activity was negatively associated with disease stage (i.e., Hoehn and Yahr) scale, as measured in the OFF (r_S_ = −0.394, *p* = 0.014) and in the ON state (r_S_ = −0.317, *p* = 0.050).

In addition, no significant correlation was found between daily-living physical activity and the number of falls, both in the PIGD (r_S_ = 0.150, *p* = 0.399) and the TD (r_S_ = 0.145, *p* = 0.462). Contrary to expectations, overall FOG severity was not associated with physical activity in the PIGD subtype (r_S_ = 0.050, *p* = 0.778) or the TD subtype (r_S_ = −0.140, *p* = 0.477). Fear of falling (represented by scores on the Activities-specific Balance Confidence Scale) tended to be associated with physical activity in the PIGD subtype significance (r_S_ = −0.281, *p* = 0.107), but this trend was not observed in the TD subtype (r_S_ = 0.069, *p* = 0.727).

## 4. Discussion

Differences in subjective activity of daily living (ADL), health-related quality of life, performance-based tests, and the quality of daily-living activity between the PD motor subtypes have been established [6,7]. The current study highlights differences in total daily-living physical activity between the individuals with PIGD and healthy controls, suggests that physical activity in the TD subtype was intermediate between PIGD and controls, and identifies factors that are related to physical activity in the PIGD subtype. Our results complement other clinically relevant differences regarding the PIGD and the TD motor subtypes and point to factors that may contribute to these differences. Moreover, the present findings demonstrate that bradykinesia, a hallmark of PD, was indeed significantly associated with the daily-living physical activity, especially in the PIGD subtype, underscoring the clinical value of this measure of activity.

Consistent with previous reports [9,10,11], we found that daily-living physical activity was moderately related to motor symptom severity (i.e., higher UPDRS III scores). This was present only in the PIGD subtype (recall Figure 2). Contrary to our expectations, higher motor severity in the ON state was only slightly more strongly associated with physical activity than in the OFF state. This may be explained by the limited influence of dopaminergic therapy on some of these symptoms [27]. Alternatively, perhaps the OFF state reduces gross motor function, while the movements provided in that period of the day play an important role in total daily-living physical activity capturing.

In contrast to the manifestations of the PIGD subtype, overall disease severity in both ON and OFF medication states and PD symptoms fluctuations appear to have mild, non-significant association with daily-living physical activity in the TD subtype subjects. There may be several possible explanations regarding the differences in physical activity between those motor subtypes. For example, previous reports indicate that people with the TD subtype have a lower annual rate of progression in the MDS-UPDRS scores [28]. Moreover, the tremor score was not associated with the severity or progression of motor complications [29] and, according to our findings, was also not related to objective daily physical activity counts. An additional explanation of the less restricted physical activity in the TD subtype could be linked to non-motor symptoms of PD. Individuals in the TD group tend to suffer less than individuals in the PIGD group from a variety of non-motor symptoms that are known to also influence physical activity [6].

However, there are some surprising findings related to the PIGD subtype too. For instance, FOG, another hallmark of disease severity of the PIGD subtype, was not directly related to the daily-living physical activity levels. This finding may indicate that symptoms like freezing of gait, fear of falling, and fall history could be mediators, secondarily related to the physical activity causing gait and balance disturbances. Poor balance performance (represented by the Berg balance test) was negatively associated with the total daily-living physical activity in both subtypes (recall Figure 2), yet more so in the PIGD individuals. Similarly, gait index scores were highly reflected in the daily physical activity, as expected. These disturbances may contribute to less consistent and less confident gait and result in a reduced physical activity among the PIGD subtype. Furthermore, patients with PD who experience FOG are more sensitive to dual-task costs during walking [30]. These hazardous conditions and other factors may alter daily-living physical activity into a high resource-consuming function (due to different levels of dual-tasking) for subjects with PD of the PIGD subtype and may cause self-restriction of physical activity.

Parts I and II of MDS-UPDRS (representing self-report of ADL difficulties and non-motor symptoms) are often used to evaluate therapeutic effects in clinical trials, to track longitudinal changes in PD cohorts over time, and to guide clinical decision-making. Previous studies have demonstrated varying results on the agreement of MDS-UPDRS Part II with objective measures of daily-living activity [11,31,32]. In the current work, we did not find significant associations between the self-report patient’s perspectives of non-motor and motor symptoms (i.e., scores on the UPDRS Parts I and II) and the total daily-living physical activity among the PD subtypes (recall Figure 3). Several factors may explain the differences between our and the aforementioned findings: (1) Subjects received different instructions regarding how to maintain their weekly routines [32], (2) The placement of the sensor to measure step counts as a proxy for daily-living physical activity was different than in our study [11]. Still, our results are based on collection and processing methods of total daily-living physical activity that are consistent with many previous studies that assessed daily-living physical activity [1,20,33]. Studies that used wearables to assess daily-living ambulation emphasize the importance of monitoring the many challenges that occur during daily routines, as compared to the relative sterile laboratory settings assessment. To date, there is a lack of standardization of physical activity monitoring in daily living. Nevertheless, wearables are not implemented in every clinic, thus it is not yet a common practice.

It appears that ADL difficulties do not accurately reflect the objective measurement of physical activity. This finding is consistent with other studies that examined the validity and interpretation of the MDS-UPDRS Part II due to psychometric limitations [34,35]. Nonetheless, optimal characterization of a subject’s response to an intervention or clinical trial should probably include, alongside the self-report, objective daily-living evaluation (e.g., using wearables).

### Limitations and Future Work

This study has several limitations: First, PD subjects and controls were recruited as part of different studies. To address this, all data preparation, processing, and analysis were performed in the same manner. Second, and perhaps more importantly, the control group was about 5 years older than the two PD subtypes. We addressed this limitation by controlling for age in all the statistical analysis. Still, even with this adjustment, the PIGD group was less active than the control subjects who were, on average, 5 years older, suggesting that the age difference did not explain the reduced physical activity that was observed in the PIGD patients. Third, the cross-sectional study design limits our ability to interpret cause and effect. Finally, a larger sample size would allow us to further explore the daily-living physical activity of PD motor subtypes compared to HC (e.g., classify the healthy and the PD subtypes using the input features).

Our results suggest that the amelioration of the severity of motor response fluctuations, motor complications, and lower extremity bradykinesia may lead to a reduction in daily-living physical activity in subjects with the PIGD subtype. It will be interesting to examine the impact of physical therapy/physical training programs and other types of interventions on daily-living physical activity, and whether this reduces bradykinesia and improves gait index, especially in subjects with the PIGD subtype. Furthermore, since balance impairment was associated with the amount of daily-activity in both subtypes, it may be beneficial to explore whether balance-targeted interventions enhance total daily physical activity levels among patients with PD.

## 5. Conclusions

The present results point to important differences between PD motor subtypes that set the stage for future research and for personalized treatment programs for people with PD, especially considering the positive health benefits associated with higher levels of daily-living physical activity in PD. These findings also highlight the importance of sensor-based assessment of daily-living physical activity, along with patients’ perspectives based on self-report.

## Figures and Tables

**Table 1:** Demographic, anthropometric, cognitive and disease-related measures in the "OFF" and "ON" state for the postural instability gait difficulty (PIGD) and the tremor dominant (TD) groups. Data are mean ± SD unless stated otherwise.

**Demographic/Anthropometric**	**PIGD** ***n* = 41**	**TD** ***n* = 32**	***p*-Value** **PIGD vs. TD**	**HC** ***n* = 29**	***p*-Value** **HC Compared to all PD Subjects**
Age (years)	64.8 ± 8.1	65.8 ± 11.0	0.997	70.9 ± 8.8	0.008 *
Sex (% male)	74.2	81.0	0.422	52.0	0.022
Body-mass-index (kg/m2)	27.0 ± 3.7	26.5 ± 3.3	0.066	26.89 ± 8.8	0.651
Education (years)	15.9 ± 3.7	15.2 ± 3.4	0.289	15.4 ± 3.4	0.717
Montreal Cognitive Assessment	25.43 ± 2.8	25.6 ± 3.0	0.766	26.3 ± 2.4	0.381
Mini-Mental State Examination	28.85 ± 1.7	29.09 ± 0.9	0.157	29.11 ± 1.0	0.997
**Disease-Related Characteristics**	**PIGD**	**TD**	***p*-Value**		
Disease duration (years)	5.6 ± 3.8	5.3 ± 2.9	0.937		
UPDRS Part I score **	11.87+4.9	9.95+6.6	0.033		
UPDRS Part II score	13.86 ± 6.8	13.07 ± 7.0	0.453		
UPDRS Part III score ON	32.14 ± 11.2	35.17 ± 12.9	0.273		
UPDRS Part III score OFF	37.7 ± 11.4	42.2 ± 13.8	0.115		
UPDRS III Delta (on vs. OFF) **	5.6 ± 5.5	7.3 ± 7.2	0.018		
UPDRS Part IV score	2.1 ± 3.2	1.9 ± 3.0	0.946		
Bradykinesia legs ON	6.11 ± 2.8	5.10 ± 3.0	0.068		
Bradykinesia legs OFF	7.13 ± 2.9	6.26 ± 3.5	0.163		
Hoehn & Yahr stage ON n (%)					
I	0	3 (1%)	0.015 *		
II	27 (67.5%)	25 (78.1%)			
III	13 (32.5%)	4 (12.5%)			
IV	1 (0.02%)	0 (0%)			
Levodopa equivalent daily dose (LEDD) (mg)FOG_Q ***	500.81 ± 387.90.00 (0_26)	543.85 ± 352.50.00 (0_13)	0.1160.001		
Number of falls in the last year ***	0.50 (0_15)	0.00 (0_2)	0.003		
Berg Balance Scale	53.00 ± 3.3	53.71 ± 2.21	0.194		
Geriatric Depression Scale	4.2 ± 3.15	4.19 ± 3.67	0.992		
Activities-specific Balance Confidence scale	87.07 ± 15.17	89.06 ± 14.4	0.127		

* *p*-value represents significance between the 3 groups according to Kruskal–Wallis test. ** significance between PIGD and TD groups according to Mann–Whitney Test *p* < 0.05, *** significance reached with respect to Bonferroni correction measured in the ON medication state.
